# Using phylogenetics to infer HIV-1 transmission direction between known transmission pairs

**DOI:** 10.1073/pnas.2210604119

**Published:** 2022-09-14

**Authors:** Christian Julian Villabona-Arenas, Stéphane Hué, James A. C. Baxter, Matthew Hall, Katrina A. Lythgoe, John Bradley, Katherine E. Atkins

**Affiliations:** ^a^Department of Infectious Disease Epidemiology, Faculty of Epidemiology and Population Health, London School of Hygiene and Tropical Medicine, London, WC1E 7HT, United Kingdom;; ^b^Centre for Mathematical Modelling of Infectious Diseases, London School of Hygiene and Tropical Medicine, London, WC1E 7HT, United Kingdom;; ^c^Centre for Global Health, Usher Institute, University of Edinburgh, Edinburgh, EH16 4UX, United Kingdom;; ^d^Big Data Institute, Nuffield Department of Medicine, University of Oxford, Oxford, OX3 7LF, United Kingdom;; ^e^Department of Biology, University of Oxford, Oxford, OX1 3SZ, United Kingdom

**Keywords:** phylogenetic tree topology, Lasso regression, ancestral-state reconstruction, HIV-1 epidemiology, who acquires infection from whom

## Abstract

Inferring the transmission direction between linked individuals living with HIV provides unparalleled power to understand the epidemiology that determines transmission. Phylogenetic ancestral-state reconstruction approaches infer the transmission direction by identifying the individual in whom the most recent common ancestor of the virus populations originated. While these methods vary in accuracy, it is unclear why. To evaluate the performance of phylogenetic ancestral-state reconstruction to determine the transmission direction of HIV-1 infection, we inferred the transmission direction for 112 transmission pairs where transmission direction and detailed additional information were available. We then fit a statistical model to evaluate the extent to which epidemiological, sampling, genetic, and phylogenetic factors influenced the outcome of the inference. Finally, we repeated the analysis under real-life conditions with only routinely available data. We found that whether ancestral-state reconstruction correctly infers the transmission direction depends principally on the phylogeny's topology. For example, under real-life conditions, the probability of identifying the correct transmission direction increases from 32%—when a monophyletic–monophyletic or paraphyletic–polyphyletic tree topology is observed and when the tip closest to the root does not agree with the state at the root—to 93% when a paraphyletic–monophyletic topology is observed and when the tip closest to the root agrees with the root state. Our results suggest that documenting larger differences in relative intrahost diversity increases our confidence in the transmission direction inference of linked pairs for population-level studies of HIV. These findings provide a practical starting point to determine our confidence in transmission direction inference from ancestral-state reconstruction.

## Significance

Identifying the transmission direction between individuals provides unparalleled power to understand infectious disease epidemiology. With epidemiological and clinical information typically unavailable to infer transmission direction, phylogenetic analysis of pathogen sequence data offers an alternative approach. While the success of this phylogenetic analysis varies, the reasons remain unknown. We analyze sequence data from over 100 transmission pairs for which both the transmission direction of HIV is known and detailed additional information is available. We find that easily quantifiable phylogenetic and sampling characteristics discriminate whether a phylogenetically inferred transmission direction is correct. Our analysis highlights that while phylogenetic approaches to infer transmission direction are unsuitable for individual-level analysis, such as forensic investigations, confidence in source attribution can be incorporated in population-level analyses.

Identifying transmission chains via contact tracing is a cornerstone of infectious disease control. It provides an opportunity to test potential cases, treat infections early, and break ongoing transmission. In addition, identifying the transmission direction provides essential knowledge for understanding risk factors of transmission and susceptibility (1–3), household transmission (4), geographical spread (5–7), and early pathogenesis events (8). This knowledge, in turn, informs the design and implementation of public health interventions (9, 10). Yet, inferring transmission direction is challenging. The comparison of symptom onset time or testing histories of linked individuals allows us to infer the direction. However, this method is restricted to cases with known contact histories and for whom other sources of infection can be ruled out, such as sexually transmitted infections occurring between self-reported sexual partners.

Comparing the ancestral relationship between pathogen genomes sampled from a putative transmission pair has been proposed as a method to identify the transmission direction (11, 12). Current approaches using ancestral-state reconstruction via parsimony-based algorithms, for example, have been used to identify the transmitting partner (11, 13, 14). Under this framework, the transmitting individual corresponds to the state at the root (i.e., individual A or B) after minimizing the number of state changes along the phylogeny necessary to explain the observed state distribution at the tips. For example, when there is paraphyly (i.e., when all sequences from one partner form a monophyletic cluster embedded within the pathogen population of the other partner), the monophyletic clade represents the recipient’s viral population (14).

While simulations suggest that using the topology of a phylogeny reconstructed from multiple viral sequences sampled from a known transmission pair can correctly identify the transmission direction, empirical tests of this hypothesis have varied in accuracy. For example, three studies incorrectly identified the direction of HIV transmission in 0/32 (15), 4/31 (16), and 4/36 couples (17). Therefore, we currently lack a method to determine the reliability of ancestral-state reconstruction inference to identify transmitting partners.

In this study, we evaluated under what conditions ancestral-state reconstruction correctly identifies the transmitting partner given that direct HIV transmission has been established between two individuals. First, we calculated how often ancestral-state reconstruction recapitulates known transmission history using 112 HIV transmission pairs with detailed information for which the transmission direction and multiple virus sequences are available. Next, we fit a statistical model to evaluate how epidemiological, sampling, genetic, and phylogenetic factors influence the accuracy of the inference. Third, we adjusted the statistical model to predict the likely success of identifying the transmission direction when only routinely collected data are available. This statistical approach provides a framework to understand the reliability of ancestral-state reconstruction to predict transmission direction across linked pairs, which can aid epidemiological investigations at the population level without making explicit claims about individual transmission events (which have legal and ethical implications for the individuals involved).

## Methods

### Data Curation.

We used publicly available HIV-1 sequence and epidemiological data from 112 transmission pairs (36 men who have sex with men and 76 heterosexual pairs) collated and described previously (8). Briefly, we first used the Los Alamos National Laboratory HIV sequence database (LANLdb) to find individuals annotated as either “transmitter” or “recipient” partners in a documented transmission cluster (up to February 2019). Second, we searched the original papers and used forward and backward citation chasing to find pairs within larger studies not listed in LANLdb. We then collated publicly available sequence and epidemiological data from LANLdb, GenBank, and the original studies. We only included the pairs with the following data: the recipient partner's infection time, the sampling times for both partners, and multiple unique sequences per individual at a single sampling time point. When multiple sampling time points were available per individual, we analyzed the data closest to the transmission time. Our analysis defines the transmitting and recipient partners as reported in the original studies, which for most pairs, correspond to self-reported sexual partners with known previous infection status. All data are provided in Dataset S1.

### Ancestral-State Reconstruction.

For each pair, we built multiple sequence alignments using MAFFT (18) and removed the columns from the alignment where gaps were found in greater than 25% of sequences. Then, we estimated the best-fit model of nucleotide substitution using ModelFinder (19). We evaluated nonparametric (FreeRate) (20, 21) vs. parametric (discrete gamma) approaches (22) to model the rate heterogeneity among nucleotide sites. We chose between three and five rate categories for the FreeRate models and four categories for the discrete gamma models. Using the best-fit model of nucleotide substitution, we then built maximum likelihood (ML) phylogenetic trees with IQ-Tree v2 (23) using a thorough nearest neighbor interchange search (24). We performed multiple independent tree searches—to account for the difficulties of inferring reliable phylogenies due to the low number of mutations in the sequence data of some transmission pairs—and selected the tree with the highest log likelihood.

Next, we performed ancestral-state reconstruction. First, we labeled the tips of each tree as sampled from either the transmitter or the recipient partner. Then, again for each transmission pair, *i*, we estimated the transmitter’s state probability at the root, *p_i_*, that maximized the likelihood of observing the state distribution at the tips using a joint estimation procedure (i.e., calculating the most likely state for each internal node in the tree while integrating over all the possible states in the other nodes) assuming equal rates of transition between the two states. These analyses were conducted using the R package ape (25, 26). Thus, *p_i_* is the probability that ancestral-state reconstruction correctly identifies the transmitter.

We evaluated the effect of the outgroup choice on the accuracy of probabilistic ancestral-state reconstruction identifying the known transmission direction. First, we generated a consensus nucleotide sequence for each transmission pair. This consensus was used to query the NCBI nucleotide database using BLAST and to identify closely related genetic sequences (27). Next, we excluded results that were at least 99% identical to our query (as they were likely part of the transmission cluster) and those corresponding to subsequent sampling time points of the individuals in the transmission pair. After exclusion, we selected the hit from BLAST with the smallest expect value. For our first rooting choice, we generated an alignment for each transmission pair that used this BLAST hit. Our second rooting choice was the oldest respective subtype-specific reference sequence from LANLdb. Our third rooting choice was a composite outgroup comprising the selected hit from BLAST and all four subtype-specific reference sequences from LANLdb. Under this final choice, we rooted the phylogenies using the oldest sequence from the outgroup. For further analysis, we chose the rooting strategy that most often recapitulated the known direction of the transmission pairs. The outgroup was removed from the tree in downstream analysis using the R package ape (25).

As a further sensitivity analysis, we also inferred the ancestral states of the ML trees using the most parsimonious reconstruction of the character's evolution, which instead of providing state probabilities, selects the state at the root that incurs the smallest number of state changes that are needed to observe the state distribution at the tips. For this, we used the Sankoff algorithm and the R package phangorn (28–31).

### Phylogenetic Inference of Transmission Direction.

In our base case analysis, we classified the inferred direction of transmission as “consistent” with the known transmission direction if *p_i_* > 0.5 or “inconsistent” otherwise. In a sensitivity analysis, we accounted for a third “equivocal” outcome by classifying the inferred direction of transmission for each transmission pair, *i*, as consistent if *p_i_* ≥ *t*, inconsistent if *p_i_* ≤ 1 − *t*, or equivocal otherwise. We used a relaxed threshold of *t* = 0.6 and a conservative threshold of *t* = 0.95 for this ordinal three-category outcome. For the parsimony-based approach, we classified the inferred direction of transmission as consistent if the state at the root was the transmitting partner, inconsistent if the state at the root was the recipient partner, or “both” if both partners were equally parsimonious at the root.

### Explaining the Accuracy of Phylogenetic Inference of Transmission Direction.

#### Using all data.

**Fig. 1.**
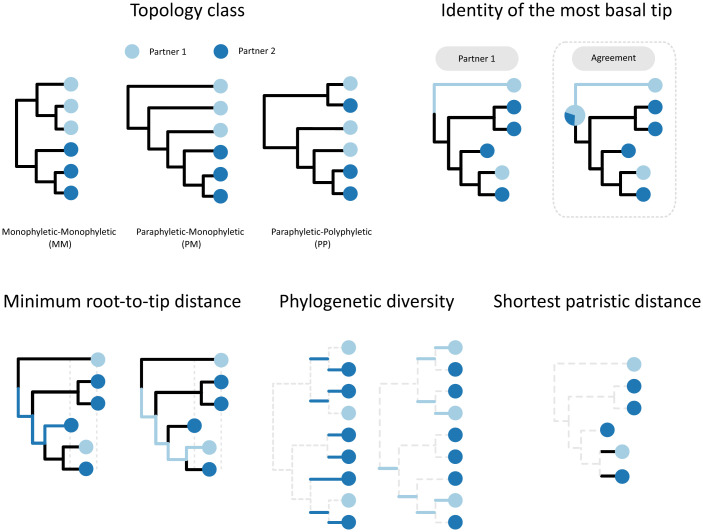
Phylogenetic covariates. Illustration of the different metrics that are used to define the covariates from the phylogenetic information class. The topology classes are PP, PM, and MM. The identity of the most basal tip is the individual with the tip that minimizes the number of internal nodes along the paths between the root and the tips (the alternative definition for inside the square corresponds to the agreement of the individual with the most basal tip with the individual with the higher probability at the root). The minimum root to tip distance is the shortest path from the root to the tips of an individual (calculated for each partner). Phylogenetic diversity indicates using the unique evolutionary history measure that is the sum of the branch lengths that are not shared across the subtree of an individual and that give rise to every single tip of the individual (calculated for each partner), as described in the documentation of the R package Caper (31). The shortest patristic distance is the shortest path connecting a tip from both individuals.

We evaluated in what circumstances ancestral-state reconstruction succeeds in identifying the transmitting partner. For this, we built a suite of logistic regression models to predict the inferred direction of transmission as a function of the information available from all transmission pairs: that is, for the base case binary outcome, the probability that the inferred direction of transmission is consistent with the known transmission direction, while for the three-class outcome, the probability that the inferred direction of transmission is consistent or inconsistent with the known transmission direction. We used 13 covariates as predictor variables that we organized into four classes: epidemiological (E), sampling (S), genetic (G), and phylogenetic (P) (Fig. 1 and Table 1).

**Table 1.** Covariates used in the two models

Information class and covariate	Values (units where applicable)	
	Model with all data	Model with routinely available data
Epidemiological (E)		
Sexual risk exposure group	Men who have sex with men or heterosexual	
Recency of the transmitter’s infection	Acute (transmission up to 90 d after infection) or chronic (otherwise)	Excluded
Sampling (S)		
Sample size	Low (no. of unique sampled sequences in either partner <10) or high (otherwise)	
Sample size difference	Difference* in the no. of unique sampled sequences between partners	Absolute difference in the no. of unique sampled sequences between partners
Time from transmission	Sum of the absolute time to sampling of both partners relative to transmission time (d)	Excluded
Genetic (G)		
Sequence alignment length	No. of base pairs	
Intrahost nucleotide diversity difference	Difference* between the within-partner mean pairwise sequence diversity (substitutions/site)	Absolute difference between within-partner mean pairwise sequence diversity (substitutions/site)
Multiplicity of infection	Single (probability of one founder unique sequence in the recipient is greater than or equal to 0.75) or multiple (otherwise)^†^	Excluded
Phylogenetic (P)^‡^		
Topology class	PP, PM, or MM^§^	
Phylogenetic diversity difference	Difference* between the sum of the branch lengths of each partner subtree^¶^ (substitutions/site)	Absolute difference between the sum of branch lengths of each partner subtree^¶^ (substitutions/site)
Root to tip difference	Difference* between the minimum root to tip distances of the partners’ sequences (substitutions/site)	Absolute difference between the minimum root to tip distances of the partners’ sequences (substitutions/site)
Most basal tip identity	Transmitter, recipient, or both; the identity of the tip(s) that minimizes the no. of internal nodes along the paths between itself and the root	Agree, disagree, or ambiguous; whether the identity of the tip(s) that minimizes the no. of internal nodes between itself and the root matches the identity with the higher ancestral-state probability at the root
Interhost patristic distance	The shortest patristic distance between tips from the partners (substitutions/site)	

^*^Subtraction of the recipient’s value from the transmitter’s value.

^†^As in ref. 8.

^‡^Illustrated in Fig. 1. To build these covariates when using a posterior distribution of trees, we selected either the most frequent observation (in the case of qualitative covariates) or the mean shift mode (in the case of the quantitative covariates).

^§^As in ref. 14.

^¶^We used the sum of the edge lengths that give rise to only one tip in the subtree as in ref. 27.

We first fitted the model with all 13 covariates from across the four classes (Table 1, all data) to identify the best set of predictors to infer transmission direction. Then, as a sensitivity analysis, we fitted an additional 14 models built from all possible combinations of these four classes: that is, four models with three classes (ESG, ESP, SGP, and EGP), six models with two classes (EG, ES, SG, SP, GP, and EP), and four single-class models (E, S, G, and P). This class-based sensitivity analysis allows us to explore the qualitative importance of different information sources to infer transmission direction.

#### Using routinely available data.

The previous suite of statistical models assumes knowledge of the transmitter and recipient’s identity in addition to epidemiological information not typically known. We developed a second suite of models with a reformulated set of eight covariates (Table 1, routinely available data) to evaluate how to interpret the inferred direction of transmission under “real-life” conditions with routinely available information.

### Model Fitting, Comparison, and Selection.

We fitted all statistical models with Lasso (least absolute shrinkage and selection operator) regression using the R packages glmnet (32) for the binary models and ordinalNet (33) for the ordinal models. Using this approach, we reduced overfitting because the Lasso regression shrinks the coefficients using a tuning parameter; these coefficients can be interpreted as evidence against the inclusion of a covariate when they shrink to zero (34). We estimated the tuning parameter using leave one out cross-validation as our dataset is small, and some of our covariate levels are uncommon—that is, there are insufficient observations to partition the data into training and validation sets. We computed ROC (receiver operating characteristic) curves to evaluate the performance of the classification models when varying *p_i_*. Then, to compare the binary models, we calculated the area under the curve (AUC) of the ROCs using the R package pROC (35). To compare ordinal models, we calculated a macro-AUC by averaging all results (one vs. the rest) with linear interpolation between points using the R package multiROC (36). We considered models with an AUC of less than 5% points apart from the model with the highest AUC to be equivalently discriminatory. Among these equivalent models, we then selected the best-fit model as that with fewer covariates (with higher AUC settling ties).

### Predicting the Accuracy of Transmission Direction Inference.

To practically assess the reliability of using ancestral-state reconstruction to predict transmission direction, we calculated the model-predicted accuracy of ancestral-state reconstruction for both the best-fit binary and the ordinal (*t* = 0.6) models across the range of their respective covariates from the original data used to fit the models. Specifically, we used the original categorical values for the discrete covariates or a range of values evenly distributed between the minimum and maximum of the original data for the continuous covariates; then, we conducted one-way and multiway sensitivity analyses for both the best-fit binary and the ordinal (*t* = 0.6) models to assess the importance of each model covariate on the probability that ancestral-state reconstruction predicts the correct direction of transmission.

## Results

### Data.

The 112 transmission pairs exhibited wide variation across all the epidemiological, sampling, genetic, and phylogenetic characteristics evaluated (*SI Appendix*, Fig. 1). Specifically, most of the transmitters were in the chronic stage at the time of transmission (101/112 pairs), and most of the transmitters were reported as heterosexual (36/112). The sample size was low (i.e., fewer than 10 sequences in the least sampled individual within the pair) in 56/112 pairs, while the median sample size difference was 5.5 unique sequences (interquartile range [IQR] = 1.00 to 12.00), and the median sum of the absolute time to sampling of both partners relative to transmission time was 173 d (IQR = 84 to 411 d).

The median sequence alignment length was 1,516 base pairs (IQR = 825 to 2,556); a total of 103/112 of the pairs had sequences that spanned the *env* region, while 9/112 spanned the *gag* region. The median difference of intrahost nucleotide diversity was 0.013 substitutions per site (IQR = 0.005 to 0.028), while 84/112 recipients’ infections were more probably seeded by a single variant.

The most frequent topology class was paraphyletic–monophyletic (PM; 63/112) followed by paraphyletic–polyphyletic (PP; 29/112) and monophyletic–monophyletic (MM; 20/112), while the median difference in phylogenetic diversity was 0.050 substitutions per site (IQR = 0.011 to 0.118), the median difference in minimum root to tip distances was 0.007 substitutions per site (IQR = 0.002 to 0.018), and the median of the minimum interhost patristic distance was 0.010 substitutions per site (IQR = 0.003 to 0.020). In terms of the most basal tip identity, the tip closest to the root (i.e., the one separated by the least number of internal nodes) belonged to the transmitter partner in 93/112 pairs, the tip closest to the root belonged to the recipient partner in 9/112 pairs, and tips from both partners were equally close to the root in 10/112 pairs.

**Fig. 2.**
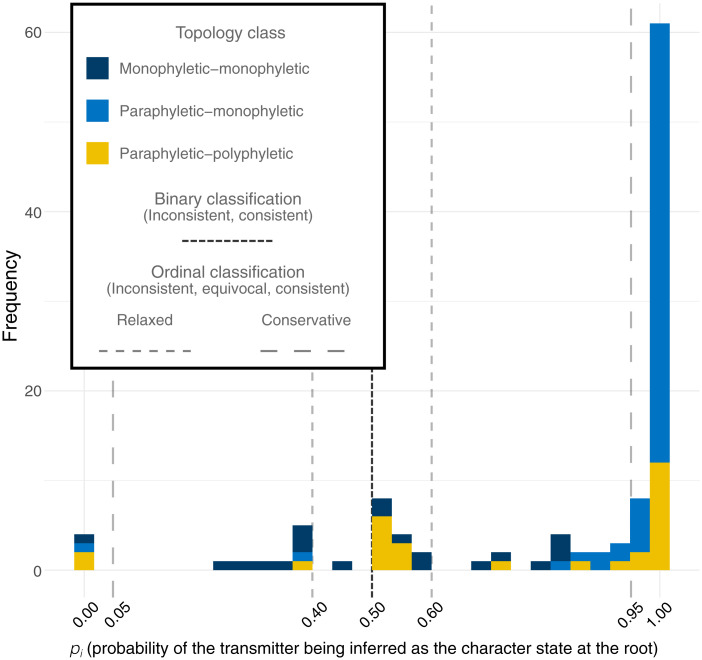
Ancestral-state reconstruction. The probability for each transmission pair, *i*, that the transmitting partner is correctly identified using ML ancestral-state reconstruction. Observations are colored by the topology class. Observations with *p_i_* > 0.5, *p_i_* > 0.6, and *p_i_* > 0.95 indicate that the inferred transmission direction was consistent with the known transmission history for the binary model, the ordinal model with relaxed threshold, and the ordinal model with conservative threshold, respectively. For the ordinal models, the outcome can be equivocal (0.4 < *p_i_* < 0.6 for the relaxed threshold, 0.05 < *p_i_* < 0.95 for the conservative threshold). The outcome is inconsistent if not consistent or equivocal.

The sample size difference, the intrahost nucleotide diversity difference, and the phylogenetic diversity difference were highly positively correlated, and they were highly inversely correlated with the root to tip difference (*SI Appendix*, Fig. 2).

### Phylogenetically Inferred Direction of Transmission.

We found that probabilistic ancestral-state reconstruction on phylogenetic trees tends to infer the transmission direction correctly regardless of the outgroup choice. When using a composite outgroup (i.e., comprising a related sequence and all four subtype-specific reference sequences from LANLdb), probabilistic ancestral-state reconstruction correctly identified the known transmission direction in 84% (94/112) of the pairs (Fig. 2 and *SI Appendix*, Table 1). This percentage was reduced to 81% (91/112) when the outgroup was a single related genetic sequence and to 80% (89/112) when the outgroup was the oldest respective subtype-specific reference sequence from LANLdb. Given these findings, we used the composite outgroup in further analyses.

There were significant differences in the topology class by outcome (Pearson’s χ^2^ *P* < 0.001). When the transmission direction was correctly inferred, the PM topology class predominated (65%, 61/94) followed by the PP topology class (23%, 22/94); in contrast, when the transmission direction was incorrectly inferred, MM (50%, 9/18) and PP (39%, 7/18) topology classes predominated.

### Explaining the Accuracy of Phylogenetic Inference of Transmission Direction.

**Fig. 3.**
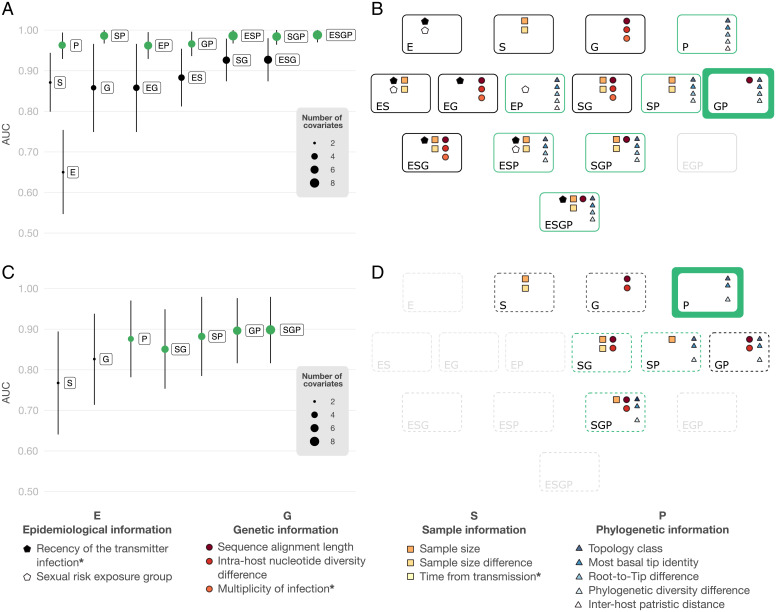
Model results. (*A*) AUC and 95% CIs of the models. The model name indicates the information’s class included in the model (i.e., epidemiological, genetic, sample, or phylogenetic). The size of each circle shows the number of covariates in the model after Lasso regression. The green color underscores the high-ranked models with equivalent discriminatory power. (*B*) The subset of covariates included in each model after Lasso regression colored by information class. The number of covariates in boxes from *B* corresponds to the size of the model in *A*. The green-colored boxes underscore high-ranked models with equivalent discriminatory power. The thick green box indicates the best-fit model. Gray-colored boxes emphasize models for which variable selection returned either a null model or a model without covariates from all the classes. (*C* and *D*) The same as in *A* and *B* but using only covariates that are routinely available and where the definition of the covariates did not consider the known direction of transmission. *Three covariates excluded in *C* and *D*.

The AUC characterizes the probability of discriminating between the correct and incorrect transmission direction. Fourteen of 16 models were kept after variable selection and regularization. We found that the 14 logistic models varied greatly in their discriminatory power to detect when the phylogenetically inferred transmission direction was correct, with mean AUC values ranging between 0.65 and 0.99 (Fig. 3*A*). There were seven models with a mean AUC greater than 0.95 and with comparable discriminatory power (the maximum ΔAUC was 0.03); these seven models all included at least four of the five covariates from the phylogenetic class (P) (Fig. 3*B*).

The model ESGP had the highest discriminatory power (AUC = 0.99, 95% CI = 0.97 to 1.00), implying that factors from all information classes affect transmission direction inference. However, when only a subset of classes is available, the model GP (AUC = 0.97, 95% CI = 0.94 to 1.00) (*SI Appendix*, Fig. 3 and Table 2) was selected as the best-fit model in accordance with our criteria. This best-fit model included four covariates of the phylogenetic class and one covariate from the genetic class. Specifically, we find that the probability of correctly inferring the transmission direction increases 1) when we observe a PM or a PP topology class (compared with MM), 2) when the most basal tip in the tree corresponds to a sample from the transmitter (compared with a tree when tips from both partners are equally close to the root), 3) when the phylogenetic diversity of the transmitter is larger than that of the recipient, 4) when the root to tip distance of the recipient is larger than that of the transmitter, and 5) when the sequence alignment length gets larger. In contrast, the probability of correctly inferring the transmission decreases when the most basal tip corresponds to a sample from the recipient (compared with a tree when tips from both partners are equally close the root).

### Predicting the Accuracy of Transmission Direction Inference Using Routinely Available Data.

#### Base case analysis.

While the previous models inform about the covariates that affect the phylogenetic inference of the transmission direction between a pair of individuals where direct transmission has been previously established, they do not inform about the accuracy of the inference when we do not know who infected whom. To tackle this issue, we reanalyzed the data after masking the identity of the transmitter and recipient and using only routinely available information. In our base case analysis (a binary model when the outcome is consistent if *p_i_* > 0.5 and inconsistent otherwise), the model fitting reduced the number of models to a total of seven that were either single-class models (S, G, P) or the multiclass models SG, SP, GP, and SGP (Fig. 3*C*). While the model SGP had the highest discriminatory power (AUC = 0.90, 95% CI = 0.82 to 0.98), model P was the best-fitting model in accordance with our criteria (AUC = 0.88, 95% CI = 0.79 to 0.97) (*SI Appendix*, Fig. 3 and Table 3), with topology class, the identity of the most basal tip, and the phylogenetic diversity difference as covariates (Fig. 3*D*). Specifically, we find that the probability that the transmitting partner is correctly identified is higher when 1) we observe a PM topology class (compared with either MM or PP), 2) the identity of the most basal tip agrees (compared with being ambiguous) with the identity of the individual with the highest probability at the root, and 3) the diversity of one partner is substantially greater than that of the other partner (the difference in phylogenetic diversity gets larger). In contrast, the probability of correctly inferring the transmission decreases when the identity of the most basal tip disagrees (compared with being ambiguous) with the identity of the individual with the highest probability at the root.

#### Sensitivity analyses.

##### Equivocal outcomes.

**Fig. 4.**
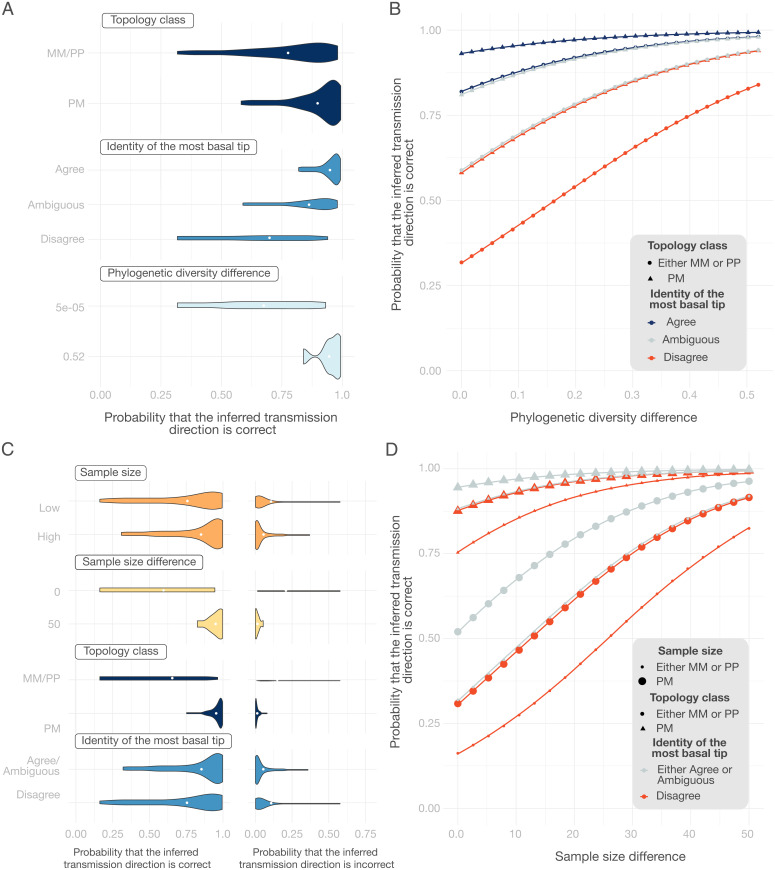
The probability that the inferred transmission direction is correct. (*A*) One-way sensitivity analysis for the binary model (consistent or inconsistent) best-fit model P, where a single covariate is fixed and all other covariates are varied over their ranges as observed in the data. (*B*) Multiway analysis with the same model in *A*, but each covariate value combination is plotted separately. (*C* and *D*) The same as *A* and *B*, respectively, but corresponding to the ordinal (consistent, inconsistent, equivocal with relaxed threshold) best-fit model SP.

When we classified the inferred direction of transmission to be consistent, inconsistent, or equivocal with a relaxed probability threshold (*p_i_* ranging between 0.4 and 0.6 as equivocal), the inferred direction of transmission was consistent with the known transmission direction in 75% (*n* = 84) of the pairs, equivocal in 13% (*n* = 15), and inconsistent in 12% (*n* = 13) (*SI Appendix*, Table 1). Similarly, using a conservative threshold (*p_i_* ranging between 0.05 and 0.95 as equivocal) increased the proportion of pairs that are classified as equivocal to 35% (*n* = 39) and reduced the proportions of consistent and inconsistent pairs to 62% (*n* = 69) and 4% (*n* = 4), respectively. The best-fit ordinal models were models SP (AUC = 0.88, 95% CI = 0.83 to 0.95) and P (AUC = 0.83, 95% CI = 0.75 to 0.90) for the relaxed and conservative thresholds, respectively (*SI Appendix*, Figs. 3 and 4 and Table 3). Similar to the base case binary model, the ordinal models show that the probability of correctly inferring the direction of transmission is higher 1) when one of the virus populations is embedded as a monophyletic group in the virus population of the partner and 2) when the identity of the basal tip either agrees or disagrees (compared with ambiguous) with the identity of the individual with the highest probability at the root. In addition, this probability increases 1) when the sample size is high and 2) when the sample size difference gets larger in the case of the SP model (relaxed threshold) or 1) when only one of the partners is very close to the root (the root to tip difference gets larger), 2) when the diversity of one partner is substantially greater than that of the other partner (the difference in phylogenetic diversity gets larger), and 3) when viral populations have not diverged much (the minimum interhost patristic distance gets smaller) in the case of the P model (conservative threshold) (*SI Appendix*, Fig. 5).

##### Most parsimonious ancestral-state reconstruction.

When we used the most parsimonious reconstruction to calculate the inferred transmission direction from the ML trees, the model P was the best-fit model (AUC = 0.84, 95% CI = 0.78 to 0.89) (*SI Appendix*, Figs. 4 and 5 and Table 3). This model suggests that the probability of the inferred direction of transmission being correct increases when 1) we observe a PM or a PP topology (compared with MM), 2) the identity of the most basal tip agrees (compared with either disagreeing or being ambiguous) with the most parsimonious state at the root, 3) the diversity of one partner is substantially greater than that of the other partner, and 4) viral populations have not diverged much.

### Implications for Bias within Population Studies.

We next evaluated whether routinely undisclosed epidemiological characteristics are associated with the probability of correctly identifying the direction of transmission. Specifically, we found that the transmitter’s infection stage at the time of transmission is associated with the topology class of the phylogenetic tree. Paraphyletic–polyphyletic topologies—which are associated with less chance of accurately predicting the transmitting partner—are more frequently observed (64%) when transmission occurred during the transmitter’s acute stage. PM topologies—that are associated with more chance of accurately predicting the transmitting partner—are more frequently observed (59%) when transmission occurred during the transmitter’s chronic stage (Pearson's χ^2^ test *P* < 0.015).

### Implications for Inference of Transmission Direction.

Our analysis suggests that transmission pair characteristics influence the likelihood of correctly identifying the transmission direction using ancestral-state reconstruction. To estimate the practical importance of this result, we used the best binary and ordinal models (P and SP) under real-life conditions to predict the chance of inferring the correct transmission direction across all possible covariates by conducting one-way and multiway sensitivity analyses.

Our one-way sensitivity analysis suggests that a high phylogenetic diversity difference leads to the most considerable improvement in inference accuracy across the covariates when outcomes are classified as consistent or inconsistent (Fig. 4*A*). If equivocal outcomes are considered, sample size differences between the individuals and topology class dominate in importance (Fig. 4*C*).

If we want to choose whether the transmission direction inferred from ancestral-state reconstruction is either consistent or inconsistent, our multiway sensitivity analysis suggests that we can be at least 82% confident when the most basal tip agrees with the identity of the individual with the higher state probability at the root. If the phylogeny has a PM topology in addition, this confidence increases to 93% (Fig. 4*B*). On the other hand, if we want to choose whether the transmission direction inferred from ancestral-state reconstruction is consistent, inconsistent, or equivocal, the probability of correctly identifying the transmitting partner is at least 94% when we observe a PM topology, when the sample size is high, and when the identity of the most basal tip does not disagree with the identity of the individual with the higher state probability at the root (Fig. 4*D*).

## Discussion

We have combined empirical data on well-characterized HIV transmission pairs with statistical modeling to determine the conditions under which ancestral-state reconstruction correctly infers the direction of HIV transmission. Our results suggest that while ancestral-state reconstruction correctly identifies the transmission direction in the majority of known transmission pairs, this success is determined by the epidemiological, sampling, genetic, and phylogenetic characteristics of the individuals and their viral populations. We show that topological and branch-length metrics—such as phylogenetic diversity differences—from the phylogenetic tree of the transmission pair affect the chances of successfully inferring the transmission direction.

To guide future work on identifying the HIV transmitting partner within a linked pair, we quantified the probability of correctly inferring the transmission direction as a function of available information. Under these circumstances, a PM topology and a match between the identity of the tip closer to the root (i.e., the one separated by the least number of internal nodes) and the identity of the state assigned to the root were highly predictive of inferring the correct transmission direction. This result agrees with the theoretical prediction that when multiple viral sequences per individual are available, the relative ordering of sequence clusters from the two individuals determines transmission direction inference (14). Practically, this means that when samples from partner A are embedded as a single clade within samples of partner B, leading to ancestral-state reconstruction commonly inferring B transmitted to A, it is highly likely (although not certain) that this inference is correct. Nevertheless, our results suggest that using a relative metric of the difference in intrahost diversity between the partners further improves discriminatory power (with larger differences indicative of a greater chance of correctly identifying the transmission direction), which is also consistent with previous work (15, 16). That is, for the example above, as the phylogenetic diversity difference between samples A and B increases, so does the confidence that B transmitted infection to A, in this case rising from 93 to 99%.

While we found several combinations of covariates that provide comparable discriminatory power, our conclusion is largely insensitive to the exact details of these models; that is, topology class, the most basal tip identity, and a measure of interhost diversity are the important drivers of accurately inferring transmission direction. This result is true when we extend our analysis across different outcomes: for example, including an equivocal class in addition to consistent and inconsistent.

There is a noticeable drop in discriminatory power when our models only include readily obtainable information, and thus, our results confirm that inferences about directionality entail considerable uncertainty in the absence of key epidemiological information (37). Moreover, because the stage of the transmitting partner’s infection is likely to influence the topology class of the phylogenetic tree, which in turn, influences the probability of correctly identifying the transmitting partner, chronic-stage transmitters who are more likely to exhibit PM topologies are thus more likely to be correctly identified (8). This argument explains, to some extent, the greater success in the transmission direction inference of recent studies from long-term follow-up serodiscordant couples, where transmission was likely to occur during the transmitter’s chronic stage (13, 15).

Our results suggest that there is little difference in the ordinal classification performance of ancestral-state reconstruction methods (either probabilistic or parsimony-based algorithms). However, we show that even when we are conservative about attributing the transmission direction using ancestral-state reconstruction, requiring a probability equal to or greater than 0.95 to support a direction, the sensitivity of ancestral-state reconstruction to determine transmission direction is not perfect at around 96%. These findings further underscore that individual-level analysis of HIV-1 transmission is not recommended and that phylogenetic analysis cannot alone prove the direction of HIV transmission between two linked individuals. In addition, a recent study tested whether the prediction of the transmission direction could be improved by using next-generation sequencing, and in the best-case scenario, the prediction was incorrect for 4/33 (12%) pairs (16). Thus, rather than focusing attention on the accuracy of the transmission direction inference for specific transmission pairs, we suggest that epidemiological studies should instead first calculate the a priori confidence in direction inference, similar to the probabilities presented here. These confidence probabilities can then be integrated directly into subsequent epidemiological analysis. Our study suggests that these confidence levels—that is, the probability of ancestral-state reconstruction correctly identifying the transmitting partner—can be easily calculated from readily obtainable sample and phylogenetic measures. For example, these probabilities could be used as weights to adjust population-level analysis, for instance, in studies investigating factors driving HIV epidemics, which rely on transmission direction.

This study has some limitations. First, well-characterized transmission pairs are scarce, and we could not test our models out of sample. Second, it is difficult to assess if there is something unique to a population consisting of transmission pairs with known transmission direction (compared to the general population where we do not know the transmission direction), which could limit the generalizability of our findings. However, our results might generalize to sexually acquired HIV-1 infections whose characteristics overlap with the range of covariate values we documented here. Third, we used relational metrics that summarized the magnitude of differences in the intrahost diversity (i.e., the difference in the number of sampled unique sequences, the difference in intrahost nucleotide diversity, and the difference in the minimum root to tip distance), in the interhost diversity (i.e., the difference in the minimum patristic distance), or in a composite measure of diversity (e.g., the difference in phylogenetic diversity). However, there are alternative ways to conceptualize diversity, and there may be other factors that affect the correct inference of transmission direction. Fourth, we did not consider the effects of processes such as superinfection and recombination, which impact diversity and phylogenetic interpretation. Fifth, we used sequences generated via capillary or Illumina sequencing of PCR products. Thus, the generalizability of our metrics when using different protocols, technologies, and next-generation sequence data warrants further investigation. Finally, we considered only pairs of individuals for whom direct transmission had been verified using information, such as contact tracing and testing histories, as detailed elsewhere. While these data are currently the best available, it is conceivable that there may have been an unsampled intermediate partner or common source partner.

Our study only addresses the chance of correctly identifying the transmission direction given that the direct transmission of infection between the two individuals has been previously established. In reality, we would also like to identify direct infection transmissions between individuals and rule out transmission through one or more intermediate partners or common sources. However, this latter question is inherently more complex because not only is the range of possible outcomes larger but also, the data on which to fit a model are scarce.

While phylogenetic analysis to infer transmission direction has recently shown immense promise, it is unsuitable for individual pair-level studies, such as forensics, because the sensitivity of ancestral-state reconstruction, even in the most optimistic conditions, is not perfect. Here, we provide a statistical framework to help explain the factors affecting transmission direction inference and to improve the reliability of future work. We stress that while phylogenies provide rich and important information about transmission at the population level, conclusions on directionality at the individual level must be considered cautiously and with full adherence to the strictest ethical standards of data use.

## Supplementary Material

Supplementary File

Supplementary File

## Data Availability

Data are available as supporting information and in a public GitHub repository (https://github.com/Chjulian/TransmissionPairs_iDoT) (38). The data file combines information from LANLdb, GenBank and the original studies, as well as previously published work (8).
