# Supplementary material for: Using phylogenetics to infer HIV-1 transmission direction between known transmission pairs
Source: Proc Natl Acad Sci U S A. 2022 Sep 14;119(38):e2210604119. doi: 10.1073/pnas.2210604119 (PMC9499565; doi:10.1073/pnas.2210604119)
Supplement: Supplementary File [file pnas.2210604119.sapp.pdf]

Supplementary Material for

USING PHYLOGENETICS TO INFER HIV-1 TRANSMISSION DIRECTION BETWEEN KNOWN TRANSMISSION PAIRS

Christian Julian Villabona-Arenas[1,2], *Stéphane Hué*[1,2], *James Baxter*[3], *Matthew Hall*[4], *Katrina A. Lythgoe*[4], *John Bradley*[1], *Katherine E. Atkins*[1,2,3] *

[1]Department of Infectious Disease Epidemiology, Faculty of Epidemiology and Population Health, London School of Hygiene and Tropical Medicine, London, UK

[2]Centre for Mathematical Modelling of Infectious Diseases, London School of Hygiene and Tropical Medicine, London, UK

[3]Centre for Global Health, Usher Institute of Population Health Sciences and Informatics, Edinburgh Medical School, University of Edinburgh, Edinburgh, UK

[4]Big Data Institute, Nuffield Department of Medicine, University of Oxford, Oxford, UK

* Corresponding author: Katherine.Atkins@ed.ac.uk

Supplementary Table 1. Accuracy of inferred direction of transmission by method

Inferred direction of transmission	Binary	Ordinal		
	t=0.5	*t*=0.60	*t*=0.95	MPR†
Consistent	94 (83.9%)	84 (75.0%)	69 (61.6%)	82 (73.2%)
Equivocal	-	15 (13.4%)	39 (34.8%)	25 (22.3%)
Inconsistent	18 (16.1%)	13 (11.6%)	4 (3.6%)	5 (4.5%)

† Most parsimonious reconstruction

Supplementary Table 2. Details of the base-case best-fit classification binomial model with all data

**Model**	**AUC**	**Covariates** [level][†] (Shrinkage coefficient)
GP	0.966 (0.936-0.996)	Sequence alignment length (0.0002)<br>Topology class [PM] (1.642)<br>Phylogenetic diversity difference (17.021)<br>Root-to-tip difference (-67.877)<br>Most basal tip identity [Transmitter] (0.398)<br>Most basal tip identity [Recipient] (-0.498)

[†] Level for discrete covariates

Supplementary Table 3. Details of the best-fit classification models with routinely-available data

**Tree-Inference method**	**Strategy**	**Threshold**	**Model**	**AUC**	**Covariates** [level]† (Shrinkage coefficient)
Maximum Likelihood	Binary	t=0.5	P	0.878 (0.785-0.971)	Topology class [PM] (1.091)<br>Phylogenetic diversity difference (4.649)<br>Most basal tip identity [Agree] (1.152)<br>Most basal tip identity [Disagree] (-1.122)
	Ordinal	t=0.60	SP	0.876 (0.830-0.947)	Sample size [low] (-0.836)<br>Sample size difference (0.064)<br>Topology class [PM] (2.758)<br>Topology class [PP] (0.008)<br>Most basal tip identity [Disagree] (-0.887)
		t=0.95	P	0.826 (0.752-0.902)	Topology class [PM] (2.162)<br>Phylogenetic diversity difference (3.465)<br>Root-to-tip difference (1.503)<br>Most basal tip identity [Agree] (1.349)<br>Inter-host patristic distance (-11.545)
		Most parsimonious reconstruction	P	0.842 (0.781-0.888)	Topology class [PM] (3.280)<br>Topology class [PP] (1.056)<br>Phylogenetic diversity difference (2.880)<br>Most basal tip identity [Agree] (0.588)<br>Inter-host patristic distance (-6.740)

† Level for discrete covariates

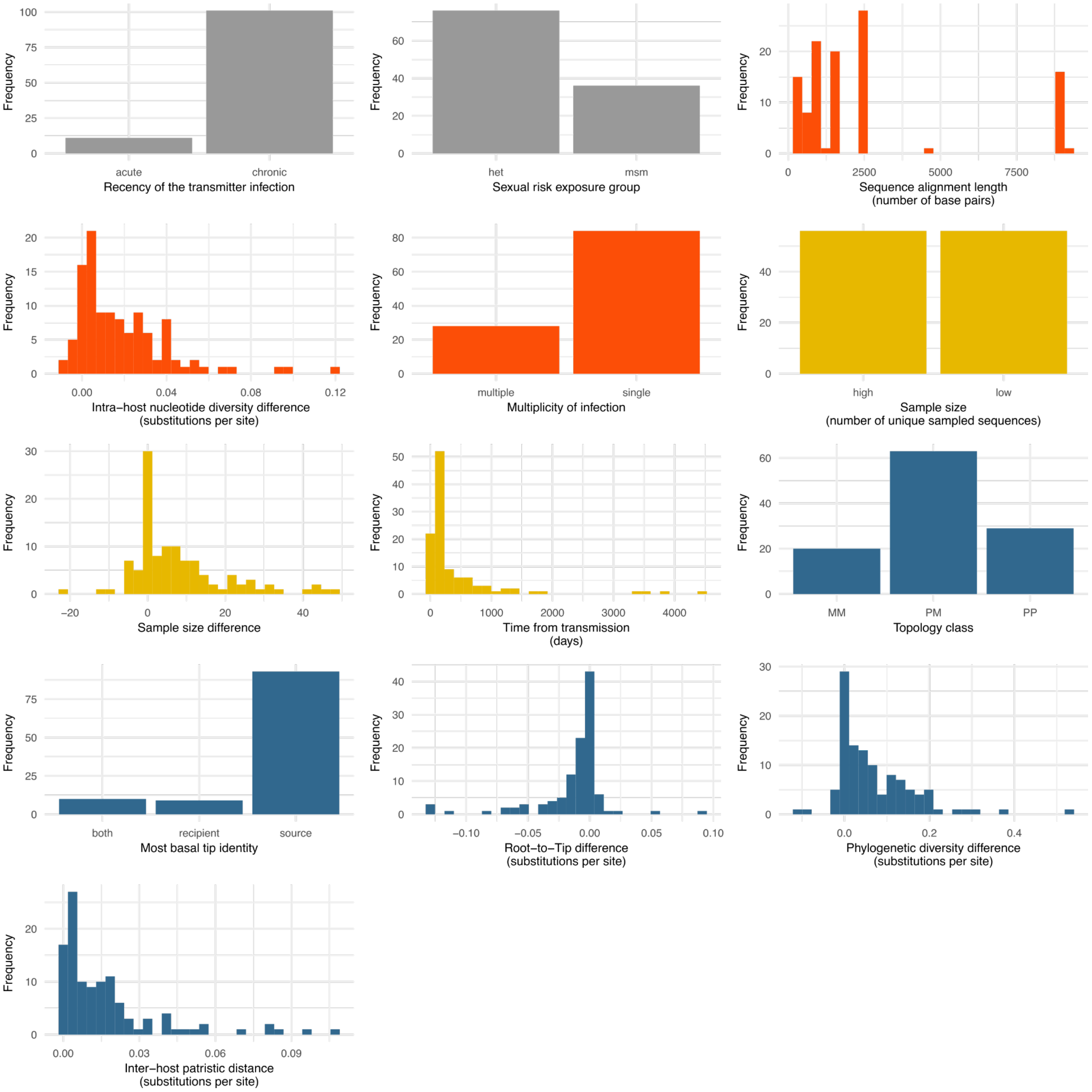

Supplementary Figure 1. Distribution of the values of the covariates. Colors indicate covariate class: epidemiological (gray), sampling (coral), genetic (dull yellow) and phylogenetic (blue)

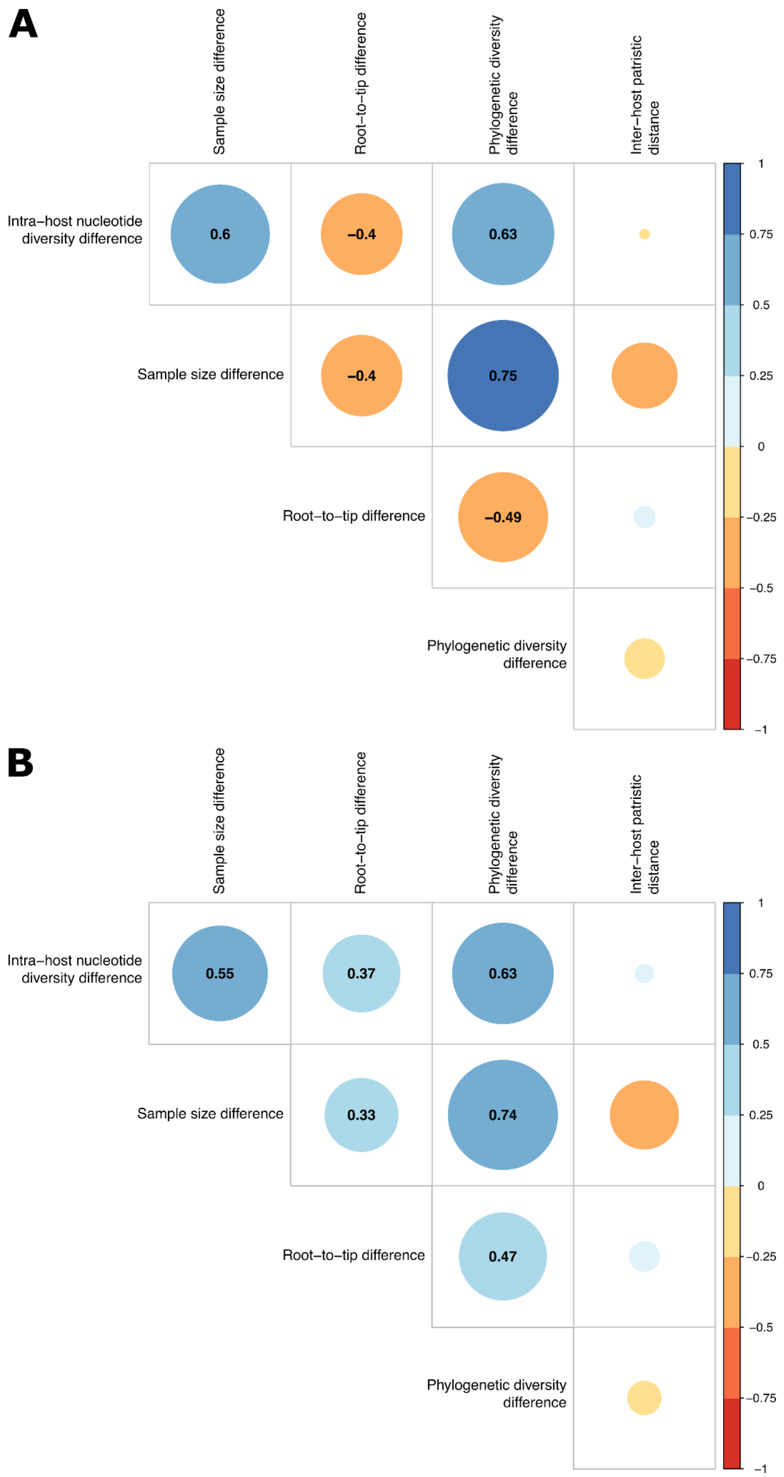

Supplementary Figure 2. Correlation matrix of the quantitative covariates. The size of each circle and the colors indicate the magnitude of Pearson's product-moment correlation coefficient. Values greater than 0.3 or lesser than -0.3 are indicated inside the circles. The coefficients were calculated using all available information (A) or ignoring the knowledge of the transmitter and recipient identity when coding the variables (absolute difference values) (B).

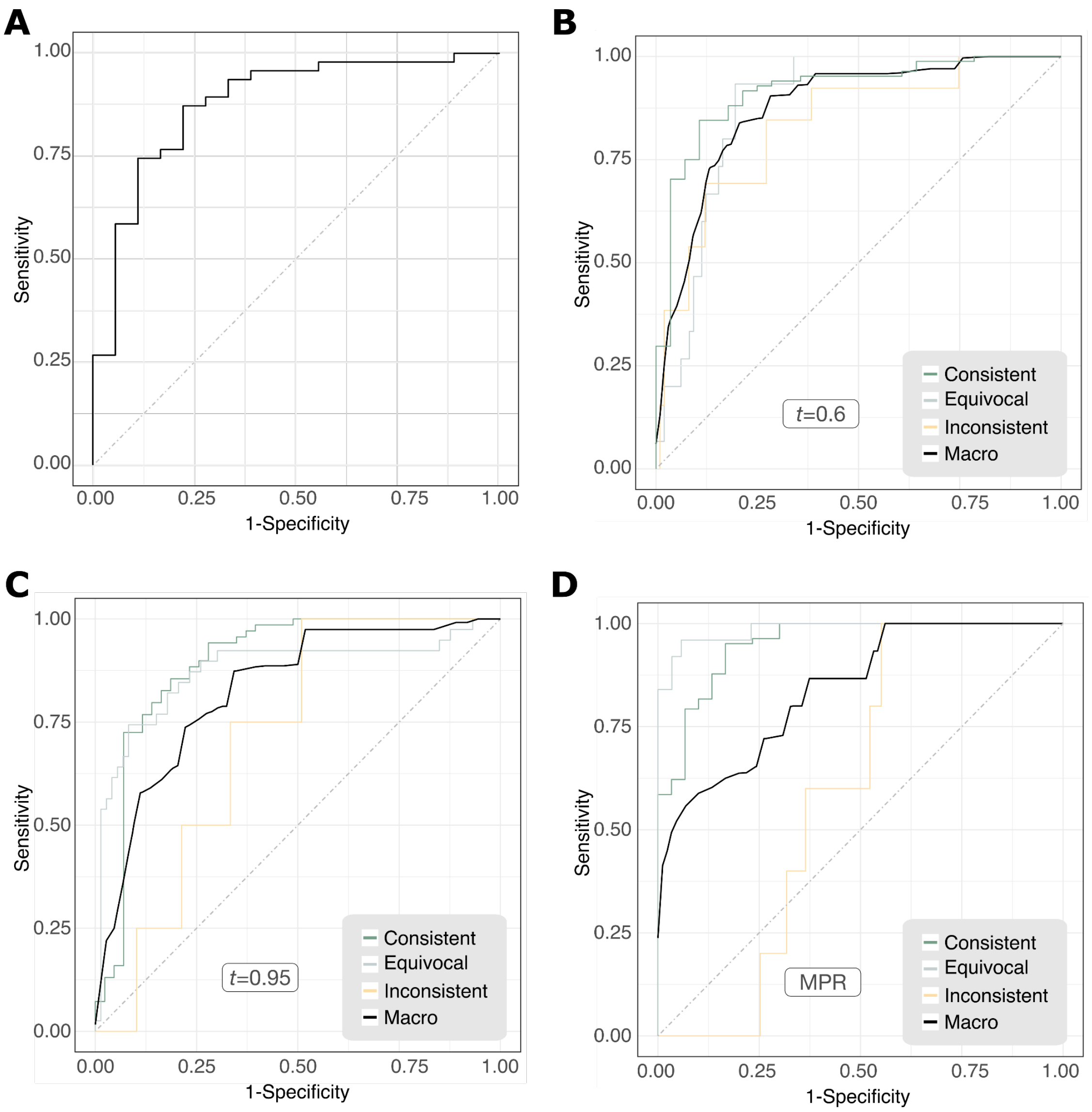

Supplementary Figure 3. Receiver operating characteristic (ROC) curves. (A) Binomial model 'P'. (B-D) Ordinal (three category) models using Maximum Likelihood (ML) or the Most Parsimonious reconstruction (MPR). The ML results are presented for the relaxed (*t*=0.60) and the conservative thresholds (*t*=0.95). The AUC of the ordinal models is represented as a one-vs-all classification (colored lines) or as the macro average (black line). Calculations given in main text.

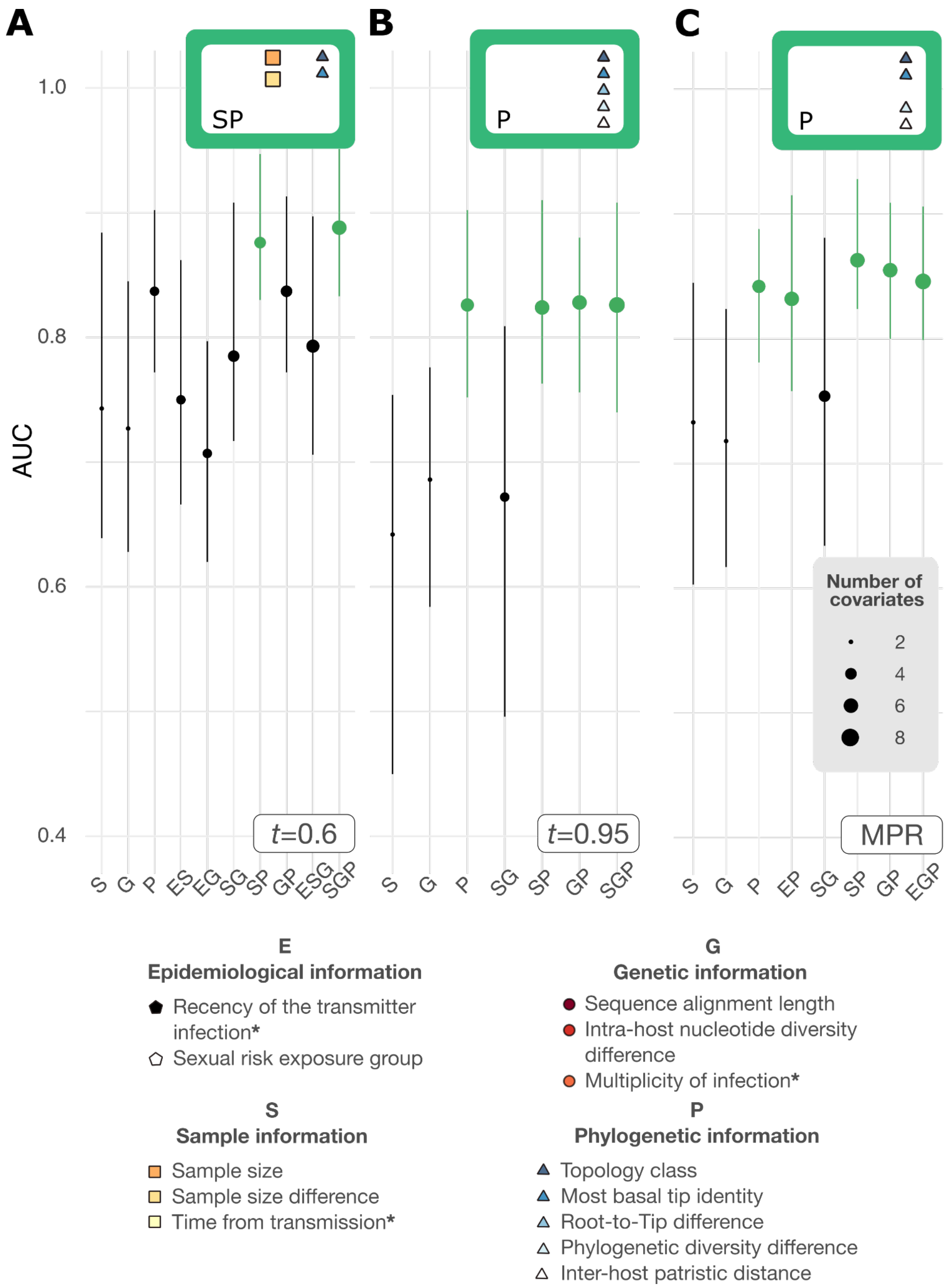

Supplementary Figure 4. Ordinal models outcomes when using routinely-available data. Macro-AUC and 95% confidence intervals of the ordinal models using Maximum Likelihood (ML) or the Most Parsimonious reconstruction (MPR). The ML results are presented for the relaxed (*t*=0.60) and the conservative thresholds (*t*=0.95). The model's name indicates the information's class included in the model (i.e. Epidemiological, Genetic, Sample or Phylogenetic). The size of each circle shows the number of covariates in the model after Lasso regression. The green color highlights the high-ranked models with equivalent discriminatory power (within 5 percentage points of the mean of the highest-ranked model) - the one with fewest covariates shown in the top boxes, indicating the model covariates; the number of covariates in the boxes corresponds to the size of the circles.

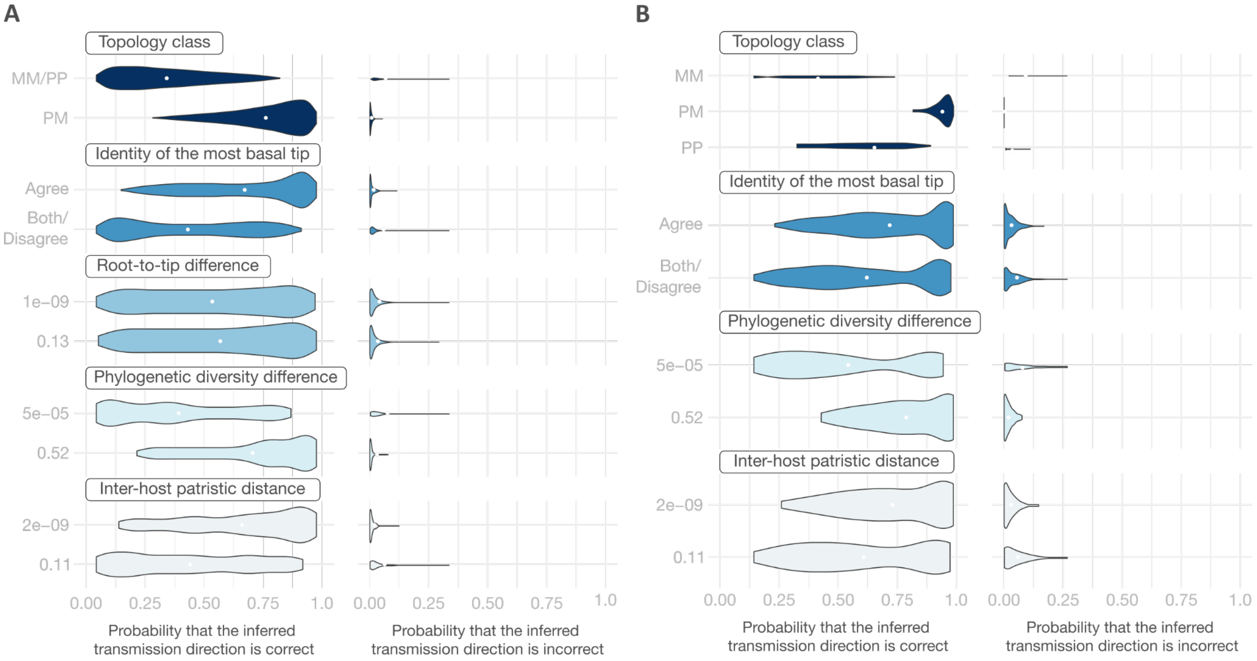

Supplementary Figure 5. Predicting the success of inferring the direction of transmission. (A) The ordinal 'P' model with a conservative threshold for the direction of transmission classification (*t*=0.95). (B) The ordinal 'P' model with Maximum Parsimony reconstruction. Units for continuous covariates are substitutions per site. The probability that the inferred transmission direction is equivocal is omitted for visualization purposes.
